# Role of Caspase-10-P13tBID axis in erythropoiesis regulation

**DOI:** 10.1038/s41418-022-01066-0

**Published:** 2022-10-06

**Authors:** Mathilde Lamarque, Emilie-Fleur Gautier, François Rodrigues, Flavia Guillem, Elisa Bayard, Cédric Broussard, Thiago Maciel Trovati, Jean-Benoît Arlet, Patrick Mayeux, Olivier Hermine, Geneviève Courtois

**Affiliations:** 1 INSERM U1163, Institut Imagine, Université Paris-Cité, Paris, France; 2 Laboratory of Excellence GR-Ex, Paris, France; 3 Institut Cochin, Département Développement, Reproduction, Cancer, CNRS INSERM UMR, 8104 Paris, France; 4 3P5 Proteom’IC facility, Université Paris-Cité, CNRS, INSERM, Institut Cochin, F-75014 Paris, France; 5 Service de Médecine Interne, Hôpital européen Georges-Pompidou APHP, Faculté de Médecine Paris Descartes, Université Paris-Cité, Paris, France; 6 Department of Hematology, Hôpital Necker Enfants Malades, AP-HP, Faculté de Médecine Paris Descartes, Université Paris-Cité, Paris, France

**Keywords:** Cell biology, Proteolysis

## Abstract

Red blood cell production is negatively controlled by the rate of apoptosis at the stage of CFU-E/pro-erythroblast differentiation, depending on the balance between erythropoietin (EPO) levels and activation of the Fas/FasL pathway. At this stage, activation of transient caspases through depolarization via mitochondrial outer membrane permeabilization (MOMP) is also required for terminal erythroid differentiation. Molecular mechanisms regulating the differential levels of MOMP during differentiation and apoptosis, however, remain poorly understood. Here we show a novel and essential role for the caspase-10-P13-tBID axis in erythroid terminal differentiation. Caspase-10 (but not caspase-8, which is activated during apoptosis) is activated at the early stages of erythroid terminal differentiation leading to the cleavage of P22-BID into P18-tBID, and later into P13-tBID. Erythropoietin (EPO) by inducing casein kinase I alpha (CKIα) expression, which in turn phosphorylates P18-tBID, prevents the generation of MYR-P15-tBID (leading to apoptosis) and allows the generation of P13-tBID by caspase-10. Unlike P15-tBID, P13-tBID is not myristoylated and as such, does not irreversibly anchor the mitochondrial membrane resulting in a transient MOMP. Likewise, transduction of a P13-tBID fragment induces rapid and strong erythroid terminal differentiation. Thus, EPO modulates the pattern of BID cleavage to control the level of MOMP and determines the fate of erythroblasts between apoptosis and differentiation. This pathway is impaired in 5q- myelodysplastic syndromes because of CK1α haplo-insufficiency and may contribute to erythroid differentiation arrest and high sensitivity of this disease to lenalidomide (LEN).

## Abstract (web summary)

Model describing the critical impact of EPO levels on P18-tBID phosphorylation through up-regulation of CK1α, which determines the BID cleavage pattern (differentiating P13-tBID or apoptotic MYR-P15-tBID) and thus the immature erythroblasts’ fate.

## Graphical abstract

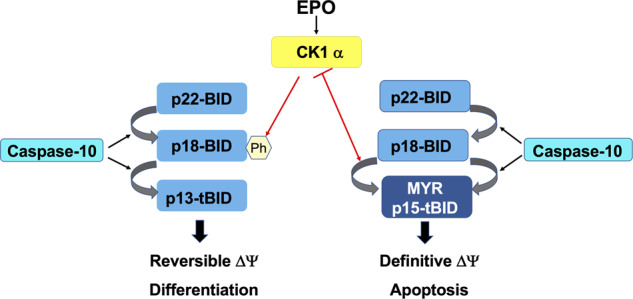

## Introduction

The production of red blood cells is a stepwise process of proliferation, survival and differentiation, from erythroid progenitors to mature red blood cells following the sequential formation of proerythroblasts, basophilic, polychromatophilic, and orthochromatic erythroblasts, which undergo enucleation, leading to the production of reticulocytes and circulating erythrocytes [1]. The positive regulation of erythrocyte production involves a combination of micro-environmental factors and cytokines, including stem cell factor (SCF) in early erythropoiesis and erythropoietin (EPO) in late erythropoiesis, which are required for cell survival and proliferation, allowing the differentiation program to occur [2]. A reduction in EPO levels leads to cell cycle arrest and apoptosis of progenitors via mitochondrial depolarization and sequential caspase-9 and −3 activation [3]. It has been suggested that the negative regulation of erythropoiesis also occurs, at another level, within the erythroid blood islands of the bone marrow as a result of interactions between erythroblasts expressing Fas and FasL leading to caspase-8 activation, differentiation arrest and apoptosis [4]. EPO levels control whether or not erythroid cells enter apoptosis or a differentiation program, although the underlying molecular mechanisms controlling this process are not yet fully understood and particularly the role of proteins of the BCL-2 family [4–6].

We and others have previously demonstrated that the activation of caspases in basophilic erythroblasts is also strictly required for terminal erythroid differentiation [7–9]. In contrast to apoptosis, a few selected caspase-3 targets, involved in structural changes observed during terminal erythroid differentiation are cleaved, while others targets, like GATA-1 the master gene of erythroid differentiation, are not [7, 10, 11]. In previous works we have shown that GATA-1 is protected from caspase-3 cleavage during erythroid differentiation by the chaperone HSP70, which localizes in the nucleus at the time of caspase activation [12–14]. In addition, in contrast to apoptosis resulting from EPO starvation, we have also shown that during erythroid cell differentiation, caspase-3 activation is transient and occurs after mitochondrial outer membrane permeabilization (MOMP) at levels that do not trigger a critical, irreversible checkpoint. The basophilic-erythroblast differentiation stage of erythropoiesis is particularly vulnerable to death at low EPO concentrations and appears to be the primary checkpoint that determines the fate of erythroblasts between terminal erythroid maturation or apoptosis [12]. The molecular mechanisms controlling this checkpoint have not been fully characterized, although BH3-only family proteins may well be involved. Among the BH3-only members, BID appears to be unique, since it connects the extrinsic pathway initiated by death receptors activation to the intrinsic pathway of mitochondria-based apoptosis, resulting in an amplification loop. Although full-length BID P22 exhibits some pro-apoptotic functions, BID apoptotic activity is fully realized by subsequent proteolytic cleavages by caspases, particularly caspase 8, which expose the hydrophobic face of the BH3 domain [15]. Death receptor ligation activates caspase-8, cleaves human BID at residue D60 to yield the typical pro-apoptotic P15 truncated fragment of BID (P15-tBID) [16]. This truncation exposes a glycine residue to N-myristoylation (Myr-P15-tBID). The P7/Myr-P15-tBID complex then targets mitochondria with increased efficiency [17] and induces the allosteric activation and homo-oligomerization of BAX and BAK, leading to the mitochondrial pore formation. BID can also be cleaved at D60 and D75 by caspase-10. In addition to cleavage, BID activity can be regulated by phosphorylation at several residues [18–20]. Phosphorylation induced by casein kinase I alpha (CK1α) and casein kinase II (CK2) decreases BID cleavage by caspase-8 in vitro [21]. CK1α preferentially phosphorylates murine residue S61, and it is likely that the two casein kinases cooperate to phosphorylate BID in vivo. It is noteworthy that caspase-10 has been highly conserved throughout evolution but is absent in rodents [22].

In the current model of erythropoiesis, EPO activates the JAK/STAT pathway and induces BCL-XL expression [23], which inhibits the MOMP induced by BH3 pro-apoptotic proteins. However, the molecular mechanisms of MOMP during differentiation or upon EPO starvation and Fas/FasL activation leading to apoptosis have not yet been fully characterized.

In the present study, we found that, during erythroid differentiation caspase-10 is activated leading to the generation of a P13-tBID fragment, whereas during apoptosis capase-8 activation leads to a P15-tBID fragment. Therefore, the differential cleavage of BID depending on the balance between Fas/FasL and EPO activation determines the fate of erythroblasts to mature or to undergo apoptosis.

## Methods

### Primary cell culture and inhibitors

CD34 + cells were isolated from umbilical cord blood, human bone marrow (Tebu-Bio reference 088SER-BMCD34-F) or mobilized PBMC. Ethical approval and informed consent were obtained (GR-Ex/CPP-DC2016-2618/CNIL-MR001). CD34 + cells were purified and mature erythroid cells were generated in a two-step amplification culture as previously described [11, 13] with the following modifications: at day 7 of the CD36 culture, 3% FBS was added, SCF concentration was decreased to 20 ng/mL and EPO increased to 3 U/mL. To activate caspase-8, the human activating, mAb anti-Fas clone CH11 (Millipore) was added at a concentration of 1 µg/mL for 20 h on day 5 of the CD36 culture, in the absence of SCF and in the presence of 0.1 or 2 U/mL EPO. To inhibit Fas signaling, both human neutralizing anti-Fas SM1/23 and anti-FasL clone 2C10 mAbs (AbCys) were added at a concentration of 10 µg/mL on day 3 of the CD36 culture in 0.5 U/mL of EPO, and were maintained throughout the culture period.

### Mitochondrial transmembrane potential assay

The MitoProbe DILC_1_(5) assay kit (Thermo Fisher) was used. Each day, a sample of 200 µl of the CD36 culture medium was incubated with 1 µl (10 µM) of DILC_1_(5). After a 30 min incubation at 37°, cells were centrifuged and labelled with PE-annexin V or eFluor 450-annexin V (according to the manufacturer’s instructions), and then analyzed on a cytometer after the exclusion of debris.

### Culture conditions of UT7 cells

UT7 cells were cultured in alpha-MEM supplemented with 10% FBS and 1% PS in the presence of 5 ng/mL GM-CSF or 2 U/mL EPO. For CK1α expression studies in UT7 cells, cells were starved for 9 h. in IMDM Iscove’s medium in the presence of 0.5% BSA, and then stimulated for 15 h. with 100 ng/mL SCF or 20, 2, 0.1, or 0.01 U/mL EPO.

### Plasmid constructs and Lentiviral production

The plasmids used in the present study were manipulated in accordance with established procedures (using DH5α cells). Oligonucleotide used for cloning and shRNA sequences are listed in Supplementary information.

Lentiviral production was previously described [13, 14]. Rescue experiments were performed by co-transducing erythroid cells on day 4 of the CD36 + cells culture with shRNA targeting CSNK1A1-pLKO-GFP vector or a control, plus BID constructs in pLVX-EF1-IRES-mCherry vector.

### Apoptosis detection and flow cytometry assays

In apoptosis assays, cultured erythroid cells were washed and labeled using the annexin V- eFluor 450 apoptosis kit, the annexin V-APC apoptosis kit or the annexin V-PE apoptosis kit (eBioscience), according to the manufacturer’s recommendations. For flow cytometry differentiation analyses, cells were washed with PBS at the indicated time and then stained for KIT/GPA expression with 1:30 human APC-anti-CD117 (eBioscience), 1:10 human PE-anti-235a (Beckman Coulter),1:20 human APC-anti-235a (Miltenyi Biotec) or 1:20 human Vioblue-anti-235a (Miltenyi Biotec). Live GFP + or CFP + cells were analyzed. For CD49d/Band3 expression, cells were stained with 1:100 human PE-BRIC6 (IBGRL Research) and 1:20 human APC-anti-CD49d (Beckman Coulter). Cells were washed with PBS and labeled with the annexin V-eFluor 450 apoptosis kit, and GFP + /annexin V- cells were analyzed for differentiation markers. FACS experiments were conducted on a Beckman Coulter Gallios or BD Fortessa Flow cytometer. The data were analyzed using FlowJo software (version 10.0.8, TreeStar)

### Detection of intracellular CK1α

UT7 cells were washed with PBS, labelled with the Fixable Viability Dye eFluor780 at a dilution of 1/1000 for 30 min at 4°, washed twice with PBS, and then fixed and permeabilized with the Foxp3/Transcription Factor Staining Buffer Set according to the manufacturer’s recommendations. After two wash with PBS, cells were stained for 30 min with FITC-conjugate rabbit anti-CK1α at a concentration of 2 µg/10^6^ cells (Assay Pro) or FITC conjugate rabbit IgG isotype control. Cell were washed and then run on a Beckman Coulter Gallios cytometer. Live cells were analyzed using FlowJo software (version 10.0.8, TreeStar).

### Mass spectrometry

The Flag-tag was introduced at the C-terminal end of the BID sequence, and cloned in the pLVX-EF1-IRES-ZsGreen vector. BID-Flag was transduced into erythroid cells, and live BID-Flag-GFP + /annexin V negative cells were sorted on day 5 and day 7 of the CD36 culture. Lysates were immunoprecipitated with Anti-FLAG M2 Affinity Gel (Sigma Aldrich), and BID-Flag proteins were separated on a 14% polyacrylamide gel. Gel regions corresponding to the migration of proteins with molecular masses of 13 and 18 kDa were cut out by hand.

### In vitro cleavage of BID by caspase-10

Labeled BID constructs were obtained, after cloning into the pCDNA3.1 vector, by in vitro transcription/translation using the TNT Quick System T7-coupled reticulocyte kit (Promega) in the presence of L-[^35^S] methionine (PerkinElmer EasyTag™ Cat. # NEG709A) according to the manufacturer’s recommendations. For CK1α phosphorylation, ^35^S-BID constructs were incubated for 3 h. at 30 °C with indicated doses of recombinant human CK1α (ab102102) or/and 50 ng recombinant human CK2 (Millipore 14–197) in kinase reaction buffer (50 mM Tris-HCl pH 7.5, 10 mM MgCl2, 1 mM DTT, 10 μM ATP). After phosphorylation, the reaction mix was incubated with 1 u or 3 u of active recombinant human caspase-10 (ab52080) for 30 min at 37 °C as indicated (One unit of the recombinant caspase-10/a (CASP-10) is the enzyme activity that cleaves 1 nmol of the caspase substrate IETD-pNA (pNA: pnitroanaline) per hour at 37 °C). Samples were loaded on a 18 cm high acrylamide gel for a better separation.

### Detection of activated BAX and BAK1

Every day of the CD36 culture, erythroid cells were washed with PBS, labelled with the Fixable Viability Dye eFluor780 at a dilution of 1/1000 for 30 min at 4°, washed twice with PBS, then fixed with PFA 4% and permeabilized with saponin 0.1% BSA 0.5%. Cells were labelled with anti-BAX 6A7 antibody (ab5714) or anti-BAK1 TC-100 antibody (Merck Millipore) for 30 min then stained with FITC conjugate anti-mouse Ab. Live cells were analyzed for activated BAX or BAK1 using FlowJo software.

### FAM-AEVD FLICA assay

Caspase-10 activity was detected with the FAM-AEVD FLICA assay kit (Immunochemistry) according to the manufacturer’s recommendations on live cells selected with the Fixable Viability Dye eFluor780. Live cells were analyzed using Flowjo software.

### Microscopy

May-Grünwald-Giemsa-stained slides were scanned (Nanozoomer 2.0, Hamamatsu), 0.23 μM/pixel (x40 high resolution mode) and visualized with the NDPview software.

### Statistical analyses

Statistical analyses were performed with GraphPad Prism software (version 6, GraphPad Inc.). Data were expressed as the mean ± SD. An unpaired two-tailed Student’s *t*-test was applied, and the threshold for statistical significance was *P < 0.05, **P < 0.01, ***P < 0.001.

## Results

### Unraveling a new caspase-10-P13-tBID axis in erythroid differentiation

**Fig. 1:**
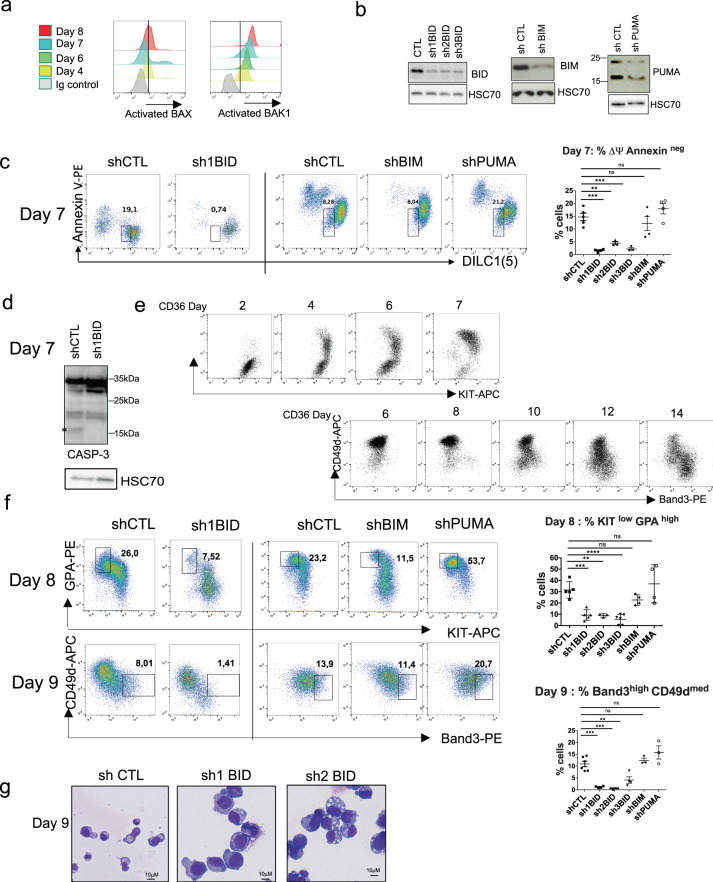
The shRNA-mediated knockdown of BID inhibits terminal erythroid differentiation. **a** Representative flow cytometry histograms gated on live cells of activated BAX (*N* = 4) and BAK1 (*N* = 3) are shown at indicated days of the CD36 culture. **b** Representative Western blot detection of BID, BIM and PUMA performed on day 4 of the culture in erythroid cells transduced with indicated shRNA (*N* = 4). **c** Representative FACS plots of GFP + cells as a function of DILC1(5) stain intensity and Annexin V performed on day 7 of the erythroid culture. The percentage of cells undergoing mitochondrial depolarization (MOMP) present in the gate is indicated. The right panel shows the percentage of cells undergoing MOMP in each experiment. The mean ± standard deviation (SD) of four replicates is shown, together with the *p*-value in an unpaired, two-tailed Student’s *t*-test) *P < 0.05, **P < 0.01, ***P < 0.001. **d** A representative western blot detection of caspase-3 performed on day 7 of the culture. (*) fully activated caspase-3 (*N* = 3). **e** Representative FACS analyses of erythroid differentiation of CD34 + cells issued from bone marrow, as a function of KIT/KPA and Band3/CD49d along the CD36 + cells culture. **f** Representative plots of erythroid differentiation of GFP + transduced cells, as a function of KIT/GPA on day 8 of the culture and of CD49d/Band3 on day 9. The percentage of more mature cells (KIT^low^GPA^high^ or CD49d^low^Band3^high^) present in the gate is indicated. Right panels show the percentage of mature cells in each experiment. The mean ± SD of replicates is shown, together with the p value in an unpaired two-tailed Student’s *t*-test. **g** Representative images of May-Grünwald-Giemsa staining (MGG) of cytospins performed on day 9 of the CD36 culture.

First, we investigated molecular mechanisms regulating transient mitochondrial depolarization (ΔΨ_m_) during erythroid differentiation of cord blood CD34 + cells. As expected, BAX and BAK1, essential effectors in MOMP, were activated at the onset of terminal differentiation (Fig. 1a). To investigate the potential role of the “activator” BH3s [24] in BAX/BAK1 activation, we used an shRNA-mediated knockdown approach with shRNAs targeting BIM, PUMA and three shRNAs targeting BID, which were transduced on day 5 of the CD34 + culture (Fig. 1b, Supplementary Fig. S7). Cyanine dye DiIC_1_(5) was used to measure the ΔΨ_m_ every day of the CD36 culture [11]. Differentiation in this culture system is asynchronous; on average, 14.5% ± 3.3 of the control cells showed a ΔΨ_m_ on day 6 or 7. Unlike BIM and PUMA, BID knockdown inhibited ΔΨ_m_ (Fig. 1c, Supplementary Fig. S1a), resulting in the inhibition of caspase-3 activation required for terminal differentiation [7], as shown by the absence of the fully active cleaved form P12-CASP-3 (Fig. 1d, Supplementary Fig. S7). Terminal differentiation is characterized by low and high expression of c-KIT and glycophorin A (GPA), respectively [25], an elevated Band3/CD49d ratio [26] (Fig. 1e) and typical morphological changes. We therefore used flow cytometry to assess c-KIT/GPA on days 6–8 of the culture, and the maturation of erythroblasts was analyzed with Band3/CD49d expression along with morphological analyses on days 8–12. ShRNAs targeting BIM and PUMA did not significantly inhibit terminal differentiation. By contrast, the mean percentage of shBID-GFP + cells committed to terminal differentiation was much lower, as was the mean percentage of mature erythroblasts (Fig. 1f, g and Supplementary Fig. S1b). In addition, erythroblasts with BID knockdown were more resistant to apoptosis induced by a FasL mimetic monoclonal antibody (CH11) (Supplementary Fig. S1d). Similar results were obtained with adults CD34 + cells culture (Supplementary Fig. S1c). Taken as a whole, these data suggest that BID is required at the onset of terminal erythroid differentiation.

**Fig. 2:**
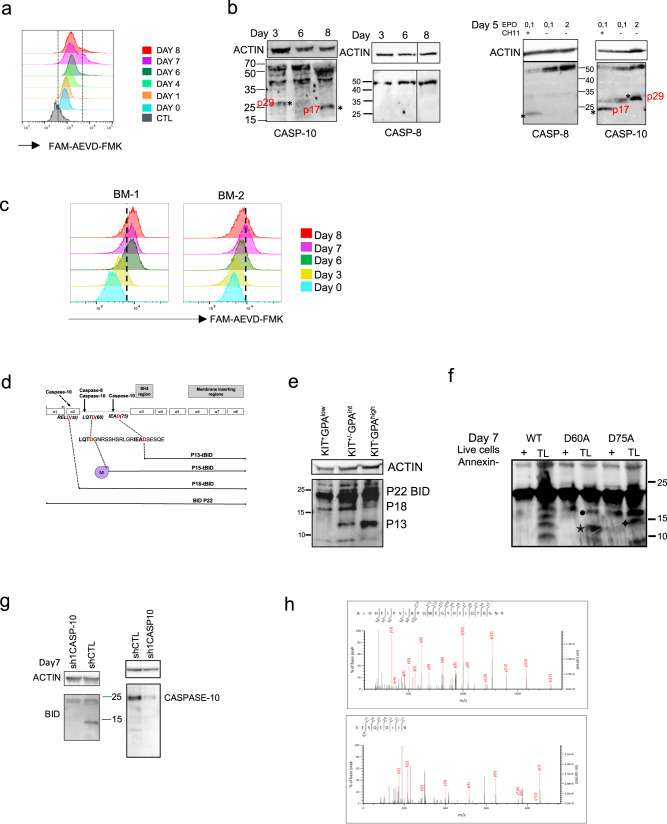
Activated caspase-10 cleaves P22-BID into P18-tBID and P13-tBID. **a** Representative flow cytometry histograms of a caspase-10 activity assay performed on living cells at indicated days (*N* = 4). **b** (Left panels) Western blots detection of caspase-10 and caspase-8 performed on sorted live cells (PI/annexin-V negative) at indicated time of the CD36 culture for expression of KIT/GPA: day 3: KIT^+^GPA^low^, day 6: KIT^+/low^GPA^int^, day 8: KIT^low^GPA^high^. (*) Caspase-10 activated forms p29 kDa and p17 kDa. (Right panels) Western blots performed on day 5 of the CD36 culture performed at several EPO concentrations in the presence or not of the Fas-L mimetic monoclonal antibody CH11. *Fully activated caspase-8 (P10 kDa), fully activated caspase-10 (P17 kDa) in addition to p29kDa. (*N* = 3). **c** Representative flow cytometry histograms of a caspase-10 activity assay at indicated days, performed on living erythroid cells cultured from bone marrow CD34 + cells (*N* = 3). **d** A schematic representation of the different BID fragments generated by caspase-8 and -10 cleavage. Cleavage at position D60 exposes a glycine to N-myristoylation (M), generating Myr-P15-tBID. A putative caspase-10 cleavage site at position D38 is indicated. **e** Representative western blot analysis of BID performed on live, sorted cells (PI/annexin-V-negative) at different time points of the culture for expression of KIT/GPA: day 2: KIT^+^GPA^low^, day 6: KIT^+/-^GPA^int^, day 8: KIT^low^GPA^high^ (*N* = 6). Cleaved forms P18 and P13 are indicated. **f** Representative western blot analyses of BID performed day 7 on: (+) live sorted cells (PI/annexin-V-negative) or (TL) total lysates of the same culture, expressing wild-type P22-BID (WT), P22-BID-D60A and P22BID-D75A. (P18-tBID (•), P15-tBID (✦), P13-tBID (✶)). (*N* = 4). **g** Western blot anti-BID and anti-caspase-10 performed on day 7 of sh1CASP10-GFP + and control transduced cells culture. **h** Semi-tryptic analysis of the mass spectrometry data (using Mascot) extracted from the 18 kDa gel area (Mascot score for the upper spectrum: 54) and the 13 kDa gel area (Mascot score for the lower spectrum: 30).

Since, as previously shown, caspase-8 is not activated during terminal differentiation we made the hypothesis that caspase-10 could be responsible of BID activation [7]. We performed a caspase-10 activity assay (Fig. 2a) and western blot analyses of sorted annexin-V-negative CD36 cells at different culture time points. Caspase-10 was activated early in the cord blood CD36 + cells culture, as shown by the presence of the CASP-10-cleaved p29 kDa fragment detectable at day 3–4 (Fig. 2b, Supplementary Fig. S2a, Supplementary Fig. S7). This p29 kDa fragment was further processed into a 17 kDa fragment at day 7-8 of the CD36^+^ culture. In contrast, we confirmed that caspase-8 was not cleaved during erythroid differentiation but as a control was activated in the presence of the CH11 antibody at low concentrations of EPO, when apoptosis occurs (Fig. 2b, Supplementary Fig. S7). Similar phenomenon was observed in adult CD34 + cells culture, in which caspase-10 activation and the onset of terminal differentiation occurred on day 6-7 (Fig. 2c)

Caspase-10 can potentially cleave BID at residue D60 to generate the apoptotic Myr-P15-tBID fragment as does caspase-8, and at residue D75 to generate P13-tBID (Fig. 2d) [22]. To analyze the pattern of BID cleavage during terminal erythroid differentiation, we performed western blot analyses of sorted live cells at different time points during the culture. A cleaved 18 kDa fragment (P18-tBID) was first detected in immature CD36 + -sorted cells. A cleaved 13 kDa fragment (P13-tBID) form then appeared at the onset of terminal erythroid differentiation. Levels of P13-tBID peaked on day 8 of the culture in more mature cells, when expression of the P18-tBID form decreased (Fig. 2e, Supplementary Fig. S7). This finding suggests that BID could be sequentially cleaved from P18-tBID to P13-tBID. To further identify the cleaved forms during differentiation, we used site-directed mutagenesis of residues D60A or D75A to inhibit cleavage by caspase-10. Western blots indicated that the 13 kDa cleaved form is absent in P22-BID-D75A lysates and the 15 kDa cleaved form is absent in P22-BID-D60A lysates (Fig. 2f, Supplementary Figs. S2b, and S7). Moreover, caspase-10 knockdown inhibited the cleavage that generates the 13 kDa fragment of BID (Fig. 2g, Supplementary Fig. S7). These data indicate that the 13 kDa fragment likely corresponds to P13-tBID. BID sequence assessment revealed another potential caspase-10 cleavage site (RELD_38_), which may generate the P18-tBID cleaved form (Fig. 2d). To confirm that BID is cleaved by caspase-10, we performed mass spectrometry. A FLAG-tagged form of P22-BID was transduced into erythroid cells, and live P22-BID-FLAG-GFP + cells were sorted on day 5 and day 7 of the CD36 culture. BID-FLAG proteins were immunoprecipitated from lysates with an anti-FLAG antibody and separated on a polyacrylamide gel. Proteins with a molecular mass of ~13 kDa or 18 kDa were digested with trypsin and analyzed by mass spectrometry. Mascot analysis of the data unambiguously identified two semi-tryptic peptides corresponding to the expected N-terminus of the BID C-terminal cleavage products generated by caspase-10 (i.e. P18-tBID, identification *p*-value < 4 × 10^−6^ and P13-tBID, identification *p*-value < 0.0011) (Fig. 2h and Supplementary Fig. S2c, d).

### Impact of the caspase-10-P13-tBID axis on erythroid differentiation

**Fig. 3:**
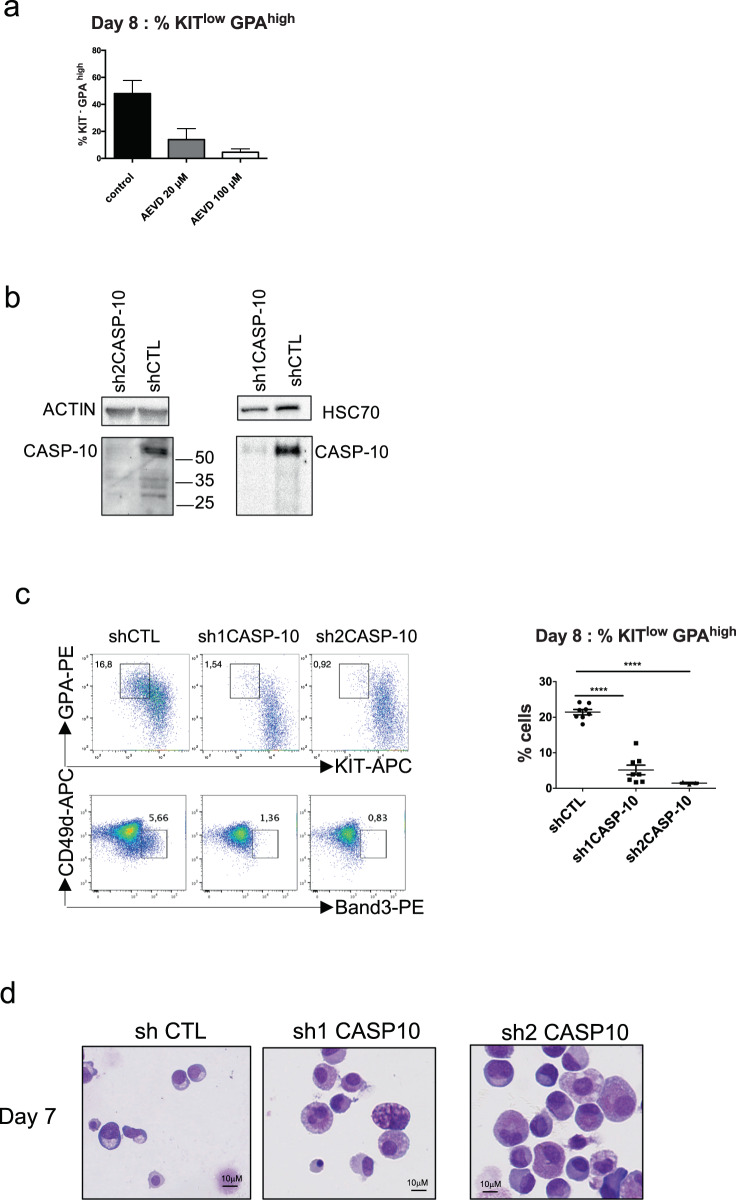
Erythroid terminal differentiation dependent on caspase-10 activity. **a** The graph shows the percentage of KIT^low^GPA^high^ cells at day 8 of the CD36 culture in the presence of the caspase-10 inhibitor AEVD-Fmk (*N* = 3). The mean ± SD of replicates is shown, together with the *p*-value in an unpaired two-tailed Student’s *t*-test. **b** Representative western blot detection of caspase-10 performed at day 7 of the CD36 GFP + sorted cells transduced at day 2 with indicated shRNA (*N* = 8). **c** Representative plots of the erythroid differentiation of transduced shCASP10-GFP + and control cells as a function of KIT/GPA and of CD49d/Band3 on day 8 of the CD36 culture. The percentage of more mature KIT^low^GPA^high^ and CD49d^low^Band3^high^ cells in the gate are indicated. The graph on the right panel shows the percentage of mature cells on day 8 in each experiment. The mean ± SD of eight replicates for sh1CASP10 and three replicates for sh2CASP10 are shown, together with the p-value in an unpaired two-tailed Student’s *t*-test. **d** Representative images of May-Grünwald-Giemsa staining of cytospins obtained on day 7 for shCTL and both shCASP10-GFP + cells, when transduction was performed on day 2 of the CD36 culture.

To investigate caspase-10 function in terminal differentiation, we first added the caspase-10 inhibitor z-AEVD-Fmk on day 0 of the CD36 cells culture. A dose-dependent decrease in terminal differentiation on day 8 was observed - suggesting that the caspase-10 activity is involved in terminal erythroid differentiation (Fig. 3a). Then, we applied a knockdown approach on both cord blood and adult CD34 + cells culture, employing two shRNAs against CASP10, sh1CASP10 and sh2CASP10 (Fig. 3b, Supplementary Fig. S3a). When transduction was performed on day 5 of the CD34 + culture, the percentage of shCASP10-GFP + cells decreased on day 4 of the CD36 + culture. Moreover, the level of apoptosis was dramatically higher on day 7 (Supplementary Fig. S3b). Transductions were then performed on day 2 of the CD36 culture. Again, an average of 50% of the shCASP10-GFP + cells were lost between days 4–7, revealing a selective disadvantage. On day 8, cell differentiation was strongly inhibited by caspase-10 knockdown (Fig. 3c, d, Supplementary Fig. S3c) and apoptosis levels were greatly elevated. These data demonstrate that caspase-10 activity is required at the onset of terminal differentiation to induce erythroid differentiation and inhibit apoptosis. To determine whether caspase-10 activation is dependent of the death receptor-mediated signal at the onset of terminal differentiation, we added blocking antibodies anti-FasL and anti-Fas to the CD36 + cells on day 3 of the culture. In favor of this hypothesis, we observed an inhibition of the transient ΔΨ_m_ and terminal erythroid differentiation (Supplementary Fig. S3d). This experiment showed that the extrinsic apoptosis pathway is involved in the onset of terminal differentiation and is in line with a previous study using siRNAs that also demonstrated that Fas/FasL (but not TRAIL) provided a positive stimulus for the maturation of human early erythroid progenitors without triggering apoptosis [27].

**Fig. 4:**
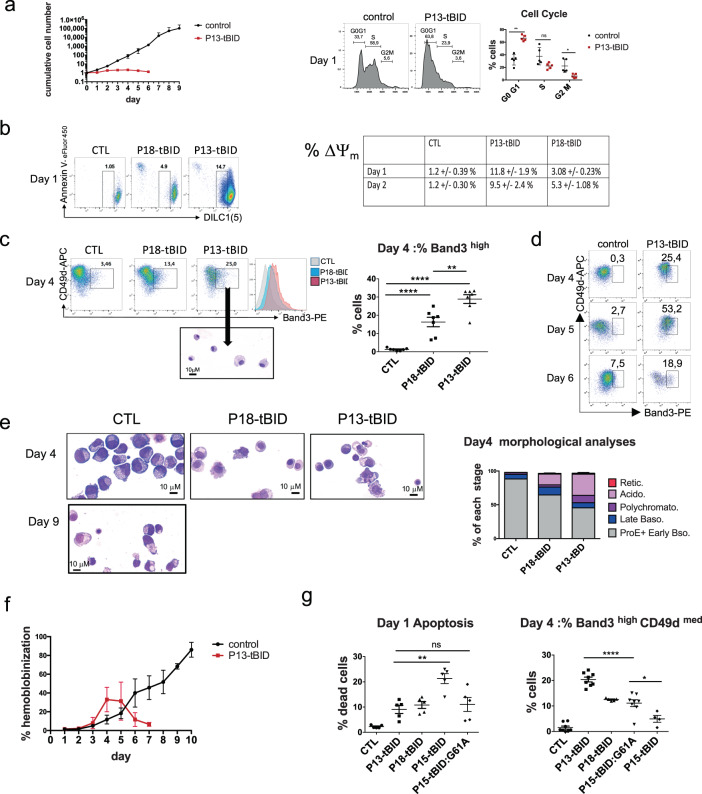
P13-tBID induces mitochondrial outer membrane permeabilization and terminal erythroid differentiation. **a** (left panel) The graph indicates the cumulative cell number assessed daily after Trypan Blue staining for each culture (*N* = 5). (Right panel) Representative cell cycle histograms performed on day 1 of the P13-tBID transduced CD36 and control cultures. The percentage of cells present in each cell cycle phase is indicated. The graph shows the percentage of cells in phase GOG1, S, and G2M in each experiment. The mean ± SD of five replicates shown, together with the p value in an unpaired two-tailed Student’s *t*-test (*N* = 5). **b** Representative FACS plots of indicated GFP + cells as a function of DILC1(5) stain intensity and Annexin V performed on day 1 of the CD36 culture. The table indicates the percentage of transduced cells undergoing MOMP on day 1 and 2 of the CD36 culture for P13-tBID and p18-tBID. The mean ± SD of five experiments is shown. **c** Representative plots of erythroid differentiation of GFP + transduced cells on day 4, as a function of CD49d/Band3. The gate indicates the more mature cells. Below: Representative images of MGG staining of cells sorted from the indicated gate. Histograms of Band3 expression are also shown. Right panel: a graph showing the percentage of mature cells on day 4 in each GFP + cell culture. The mean ± SD of seven replicates is shown (together with the *p*-value in an unpaired two-tailed Student’s *t*-test. **d** Representative plots of erythroid differentiation of GFP + transduced cells as a function of CD49d/Band3 on days 4,5 and 6. The gate indicates the more mature cells. **e** Representative images of May-Grünwald-Giemsa staining of cytospins of P13-tBID and P18-tBID transduced cells performed on day 4 of the CD36 culture (as compared with day 9 for the control cells). Graph on the right panel indicates the percentage of cells at each distinct stage of maturation counted at day 4 of the CD36 culture after MGG staining: Proerythroblasts + early basophilic erythroblasts (E.) (ProE + early Baso), late basophilic E. (late Baso), Polychromatophilic E. (Polychromato), acidophilic E. (Acido) and reticulocytes (Retic). 200 cells were counted per experiment (*N* = 5). **f** The number of hemoglobinized cells assessed by benzidine staining at various times points of the P13-tBID and control CD36 cultures. The mean ± SD of eight replicates is shown (*N* = 8). **g** Graphs showing the percentage of apoptotic cells at day 1 and the percentage of more mature cells (CD49d^low^/Band3^high^) at day 4 of indicated GFP + culture cells (*N* = 5).

To investigate the function of each BID cleaved fragment, we designed N-terminal deletion mutants of BID resulting from caspase-10 cleavage starting at residue -A_39_ and -S_76_ for P18-tBID and P13-tBID respectively. The corresponding cDNAs were transduced on day 5 of the CD34 + culture. P13-tBID inhibited cell proliferation with a cell cycle arrest in G0/G1 (Fig. 4a). P13-tBID and to a lesser extent P18-tBID induced an early ΔΨ_m_ on day 1 of the culture (Fig. 4b), as compared to day 7 for controls. P13-tBID induced terminal erythroid differentiation after 3 to 5 days, rather than the 8–10 days observed in controls. At day 4, a mean of 28.9% of Band3^high^/CD49d^low^ cells was observed, mostly exhibiting an acidophilic morphology (Fig. 4c–e). The rate of mature cells peaked at day 4 or 5 then decreased. At that time point, CD71 was down-regulated and GPA slightly up-regulated in P13-tBID-GFP + cells, suggesting an acceleration of cell differentiation (Supplementary Fig. S4a). Hemoglobinization of P13-tBID cultured cells was accelerated but not fully complete (Fig. 4f). Expression of P18-tBID also induced terminal differentiation of CD36 + cells in 3–5 days, albeit to a lesser extent than P13-tBID (Fig. 4b, c, e). Transduction of the P13-tBID construct also induced rapid and strong terminal differentiation during adult CD34 + cells culture (Supplementary Fig. S4b).The abilities to induce apoptosis/differentiation of P15-tBID and a mutated form P15-tBID-G61A that cannot be myristoylated [17] were analyzed and compared with P13-tBID. These constructs were transduced into erythroid cells at a 10–15% efficiency since more than 15% P15-tBID transduction highly increased apoptosis. The percentage of P15-tBID-GFP + cells highly decreased during the culture and surviving cells exhibited features of apoptosis as compared to P13-tBID (Supplementary Fig. S4c). Finally, unlike P15-tBID, P15-tBID-G61A-GFP + cells were able to induce some differentiation, albeit to a lesser extent than P13-tBID (Fig. 4g). Taken together, these results demonstrate that P13-tBID is the most effective form to induce ΔΨ_m_ and erythroid differentiation, without inducing apoptosis.

### Influence of the caspase-10-P13-tBID axis in erythroid apoptosis

**Fig. 5:**
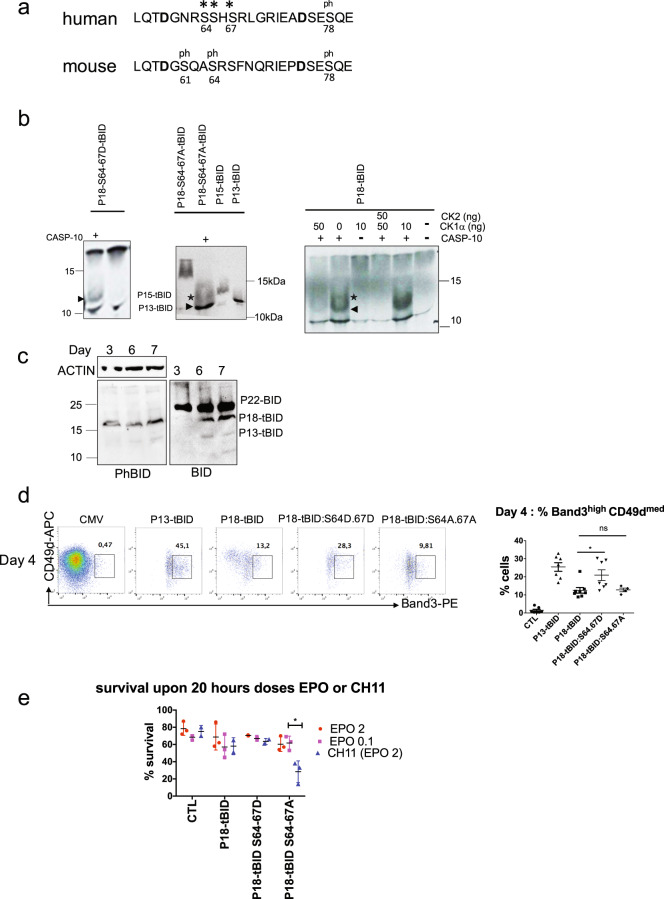
P18-tBID Serine-phosphorylation inhibits apoptosis and allows terminal differentiation. **a** Alignments of human and murine BID sequences around the caspase cleavage sites (in bold). (*) Potential phosphorylation sites in the human protein. (Ph) Phosphorylation on murine residues S61, 64, and 78 and human residue S78. **b** Representative autoradiography of indicated in vitro translated ^35^S-BID constructs loaded on a PAGE. Doses of recombinant CK1α or CK2 used before treatment (or not) with recombinant caspase-10 (+) are indicated (P15-tBID ✶and P13-tBID < ) (*N* = 4). **c** Western blot analyses of BID phosphorylation in erythroid sorted cells for expression of KIT/GPA: at day 3 KIT^+^GPA^low^, day 6 KIT^+/-^GPA^int^ and day 7 KIT^low^GPA^int^. **d** Representative plots of erythroid differentiation of indicated transduced GFP + cells, as a function of CD49d /Band3 on day 4 of the culture. The graph on the right panel showing the percentage of mature cells on day 4 in each of the GFP + cell culture. The mean ± SD of four replicates for P18-tBid:S64-67A and seven replicates for the others are shown, together with the *p*-value in an unpaired two-tailed Student’s *t*-test. **e** A graph showing the cell survival rate, after treatment with low EPO concentration or with the CH11 antibody. Three replicates are shown, together with the *p*-value in an unpaired two-tailed Student’s *t*-test for the P18-tBID-S64-76A mutant.

Caspase-10 can generate the apoptotic P15-tBID fragment, which is not observed during erythroid differentiation. We hypothesized that phosphorylation could regulate this cleavage [18–20] allowing to generate only the P13-tBID fragment. Murine residue S61 is the major site for phosphorylation by CK1α [21]. The murine S61 residue is not conserved in human BID, although human residues S64 and S67 constitute potential CK1α phosphorylation sites (Fig. 5a). Point mutations were introduced at both S64 and S67 of P18-tBID to either mimic constitutive phosphorylation (S64D and S67D: P18-S/D) or prevent phosphorylation (S64A and S67A: P18-S/A). An in vitro caspase-10 cleavage assay performed on in vitro-translated BID constructs showed that caspase-10 preferentially generates P13-tBID from P18-S/D, whereas both P15-tBID and P13-tBID are produced from P18-tBID and P18-S/A, suggesting that S64/S67 phosphorylation may protect against apoptotic cleavage (Fig. 5b, Supplementary Fig. S7). To confirm that caspase-10 cleavage at D60 is casein kinase−mediated, we performed an in vitro phosphorylation assay using different doses of CK1α or CK1α/CK2 before incubation with caspase-10 (Fig. 5b, Supplementary Fig. S7). The first caspase-10 cleavage generating the apoptotic P15-tBID fragment was inhibited by treatment with 10–50 ng CK1α, whereas higher CK1α concentrations or treatment with both CK1α and CK2 completely inhibited the caspase-10 cleavage. To study BID phosphorylation in erythroid cells, we used an antibody against the phospho-S65 residue of human BID, which mainly revealed P18-tBID phosphorylation along the CD36 culture, although phospho-P22-BID was eventually detected (Fig. 5c, Supplementary Figs. S5, and S7). To determine whether phosphorylation affects P18-tBID’s ability to promote survival or differentiation, we transduced erythroid cells with P18-S/D or P18-S/A. The S64D/S67D mutations enhanced the pro-differentiation effect of P18-tBID, while S64A/S67A mutations did not have a significant effect (Fig. 5d). However, P18-S/A-GFP + cells were progressively lost between days 1 and 4, highlighting a selective disadvantage. To further analyze survival, GFP + cells were cultured for 24 h at low EPO concentrations or in the presence of the FasL mimetic antibody CH11. Erythroblasts transduced with P18-S/D survived better than those transduced with P18-tBID under all conditions, whereas the presence of the CH11 antibody was associated with significantly lower survival of P18-S/A-GFP + cells (Fig. 5e). These results demonstrate that phosphorylation of S64 and S67 residues protects against apoptosis - likely by inhibiting the BID cleavage that generates the apoptotic Myr-P15-tBID.

**Fig. 6:**
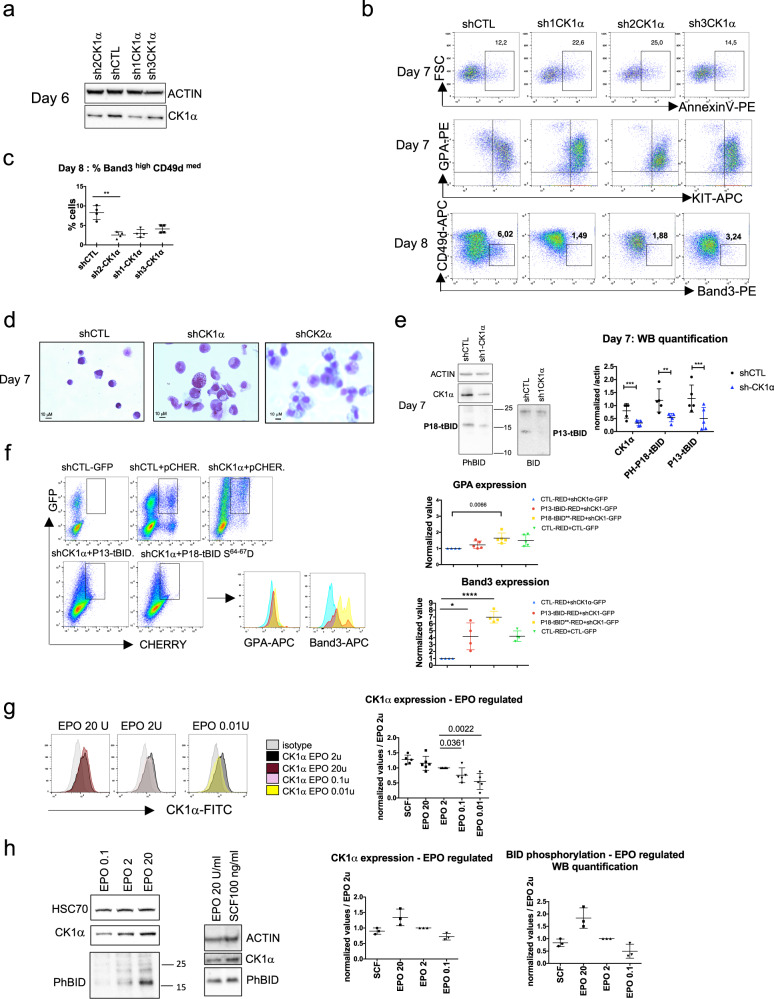
CK1α - induced P18-tBID phosphorylation is required for the inhibition of apoptosis and the progression of terminal differentiation. **a** Western blot detection of CK1α on day 6 in erythroid cells transduced on day 4 with the indicated shRNAs. **b** Three shRNAs targeting CK1α and a control shRNA were transduced on day 4 of the CD36 culture. Representative plots of apoptosis of GFP + transduced cells on day 7; the percentage of apoptotic cells in the gate is indicated. Representative plots of erythroid differentiation of GFP + cells as a function of KIT/GPA on day 7 and CD49d/Band3 on day 8. **c** The graph on the left panel indicates the percentage of mature cells on day 8. The mean ± SD of four replicates is shown, together with the *p*-value in an unpaired two-tailed Student’s *t*-test. **d** Representative images of May-Grünwald-Giemsa staining of cytospins obtained on day 7 from GFP + sorted cells. *N* = 4. **e** A representative western blot performed on sorted GFP + cells on day 7 of the CD36 culture transduced on day 4 (*N* = 4). The graph on the right panel gives the quantified intensities of the western blots. The values were normalized against actin. The mean ± SD of four blots is shown, together with the *p*-value in an unpaired two-tailed Student’s *t*-test. **f** shCK1α-GFP or control-GFP were co-transduced with indicated mCherry-vectors on day 4 of the CD36 culture, as indicated. The gate includes GFP + /mCherry +  cells which were analyzed on day 7 for GPA and Band3 expressio. A representative histogram of GPA and Band3 expression of GFP + /mCherry+ cells are shown. (Right) Graphs showing GPA and Band3 expression in co-transduced cells. Relative values are normalized against the mean fluorescence intensity for GPA or Band3 expression in shCK1α-GFP + / mCherry +  control vector co-transduced cells. The mean ± SD of five or four replicates are shown, together with the *p*-value in an unpaired two-tailed Student’s *t*-test. **g** Representative histogram plots of CK1α expression in UT7 cells cultured with different EPO concentrations. The graph on the right panel shows CK1α expression. Values are normalized against the mean fluorescence intensity for 2 U/ml EPO. The mean ± SD of five or six replicates are shown, together with the *p*-value in an unpaired two-tailed Student’s *t*-test. **h** Representative western blots showing CK1α expression and P18-tBID phosphorylation in UT7 cells. The graph gives the quantified intensities of the western blots (*N* = 2).

To confirm the role of CK1α as an inhibitor of apoptosis in terminal erythroid differentiation, three shRNAs were used to down-regulate CK1α expression (Fig. 6a, Supplementary Fig. S7). When the cells were transduced on day 5 of the CD34 + culture, ∼75% of the shCK1α-GFP + cells were apoptotic on day 4 of the CD36 culture and 100% were dead on day 6. Apoptosis also occurred on day 6 when transduction was performed on day 2 of the CD36 culture and GPA expression was still low at that time (Supplementary Fig. S6a). Lastly, transduction with the three shRNAs was performed on day 4 of the CD36 culture. Again, apoptosis was higher on day 7 (Fig. 6b) and the GPA^med^KIT^low^ cell population disappeared (Fig. 6b). This finding suggested that the cells had undergone apoptosis, whereas the more mature population GPA^high^KIT^low^ was probably generated from cells having already committed to terminal differentiation prior to CK1α knockdown. FACS and morphological analyses on day 8 confirmed that CK1α knockdown inhibited the terminal erythroid differentiation (Fig. 6b–d). Western blot analyses of GFP + cells sorted on day 7 of the culture showed lower levels of P18-tBID phosphorylation in shCK1α cells and low P13-tBID expression (Fig. 6e, Supplementary Fig. S7), suggesting CK1α is involved in P18-tBID phosphorylation and required for the P13-tBID-generating cleavage. Erythroid terminal differentiation of adult CD34 + cells was also inhibited by CK1α knock-down that induced apoptosis (Supplementary Fig. S6c). To determine whether P13-tBID or P18-S/D could rescue the CK1α defect for survival or differentiation, co-transduction of shCK1α-GFP and P13-tBID-mCherry or shCK1α-GFP and P18-S/D-mCherry was performed on day 4 of the CD36 culture (Fig. 6f). GPA and Band3 expression analyses of GFP + /mCherry+ cells on day 6 and 7 showed that P18-S/D rescued the CK1α defect more effectively than P13-tBID. This result suggests that the differentiation arrest in shCK1α-GFP + cells is associated with apoptosis, and that a constitutively phosphorylated P18-tBID may compete with its endogenous counterpart.

Given that negative regulation of terminal erythroid differentiation occurs at low EPO concentrations at the time of c-KIT downregulation, we examined whether CK1α expression and the resulting P18-tBID phosphorylation are regulated by levels of EPO or SCF. To this end, we used UT7 cells that proliferate in response to EPO or SCF and can be starved of serum and growth factors for 9 h without dying. CK1α protein expression was quantified by FACS (Fig. 6g) and western blotting (Fig. 6h, Supplementary Fig. S7) after 20 h of stimulation with SCF or at different EPO concentrations. The presence of SCF and high EPO levels was associated with elevated CK1α expression relative to cells cultured at 2 U/mL EPO. In contrast, low EPO concentrations were associated with lower CK1α expression. As a consequence, P18-tBID phosphorylation was lower with low EPO levels than with 20 U/mL EPO and 100 ng/mL SCF. These results demonstrate that P18-tBID phosphorylation depends on CK1α expression that is induced by SCF and EPO. The results also suggest that in basophilic erythroblasts, which down-regulate c-KIT, P18-tBID phosphorylation is only dependent on the EPO concentration.

## Discussion

In a simplified model of erythropoiesis, red blood cell homeostasis relies on a balance between EPO promoting cell survival and Fas/FasL inducing cell death [4, 27, 28]. Here, we show that human erythroid terminal differentiation requires activation of caspase-10 but not caspase-8, which is involved in apoptosis. In agreement with our hypothesis, a study in HeLa cells found that caspase-10 promotes cell survival by reducing the recruitment and processing of caspase-8 in the DISC [29]. However, the mechanism that determines the choice between caspase-8 or -10 activation at the DISC after Fas activation has yet to be identified.

We used mass spectrometry to identify the cleaved form P18-tBID, generated at the putative RELD_38_ caspase-10 cleavage site, which had not previously been described. Although this BID fragment has never been detected by in vitro caspase-10 cleavage assays [22], the cleavage could be dependent on specific in vivo BID modifications [30]. We demonstrated here that P18-tBID has a critical, CK1α phosphorylation-dependent role in the determination of immature erythroid cell fate between terminal differentiation and apoptosis. The *CSNK1A1* gene encoding CK1α is located in the region deleted in myelodysplastic syndrome MDS 5q-, which is associated with refractory anemia [31]. The associated impairment of erythropoiesis is characterized by amplification of the pathologic clone and a differentiation arrest at the immature erythroblast stage. CK1α is one of the targets of LEN, a highly effective treatment for MDS 5q- [32, 33] that induces apoptosis of these cells, allowing restoration of cells not bearing this abnormality. We demonstrated here that CK1α inhibition induces the apoptosis of immature erythroblasts and inhibits their terminal differentiation by decreasing P18-tBID phosphorylation and thus P13-tBID production. This mechanism might be involved in the erythroid differentiation arrest in MDS 5q-. It might also explain why erythroid cells with haplo-insufficient CK1α expression are more sensitive than normal cells with two copies of the gene to the effects of LEN, which induces the partial proteasomal degradation of CK1α [33] and apoptosis of the pathological clone.

Lastly, we demonstrated for the first time that the truncated P13-tBID fragment allows terminal erythroid differentiation by inducing a reversible mitochondrial depolarization. Unlike P15-tBID, P13-tBID is not myristoylated; this might prevent its anchorage into the mitochondrial outer membrane and could explain the transient nature of its effects. However, due to the full erythroid differentiation in addition to morphological changes, one cannot rule out the possibility that P13-tBID also induces metabolic changes critical for erythroid differentiation [34–36]. In this regard, the loss of MTCH-2 (a receptor-like protein for BID at the outer mitochondrial membrane) results in a switch from glycolysis to oxidative phosphorylation [37, 38]. Such a switch has been linked to the stem-to-progenitor cell transition [39], whereas glucose and glutamine metabolism regulate the commitment of hematopoietic stem cells to the erythroid lineage [40].

In conclusion, our findings provide evidence of a novel and complex mechanism by which EPO regulates erythropoiesis involving Fas/Fas-L, caspase-10, CK1α and BID. The differential cleavage of BID constitutes a new checkpoint that determines the fate of erythroblasts (i.e., maturation or apoptosis). It will be interesting to further investigate whether the control of caspase activation by the FAS activation/EPO level balance is disturbed in some types of anemia with ineffective erythropoiesis; this situation might provide new opportunities for treatment by selectively inducing the apoptosis of abnormal erythroid cells. Lastly, other cell differentiation systems in which caspases are activated [41] might involve similar regulatory mechanisms.

## Supplementary information

Supplementary informations

supplementary Fig S1

supplementary Fig S2

supplementary Fig S3

supplementary Fig S4

supplementary Fig S5

supplementary Fig S6

supplementary Fig.S7 - uncropped western blots

check list

## Data Availability

All data supporting the results reported here are available in the article and Supplementary files or from the corresponding author.
