# Supplementary material for: Role of Caspase-10-P13tBID axis in erythropoiesis regulation
Source: Cell Death Differ. 2022 Oct 6;30(1):208–20. doi: 10.1038/s41418-022-01066-0 (PMC9883265; doi:10.1038/s41418-022-01066-0)
Supplement: Supplementary file 1 — Supplementary informations [file 41418_2022_1066_MOESM1_ESM.docx]

**SUPPLEMENTARY INFORMATION**

**Supplemental Figure Legends**

**Supplementary Figure 1. The shRNA-mediated knockdown of BID inhibits terminal erythroid differentiation**

**(a)** Representative FACS plots of GFP+ transduced cells as a function of DILC1(5) stain intensity and Annexin-V performed on day 6 of the cord blood erythroid culture. The percentage of cells undergoing mitochondrial depolarization (MOMP) present in the gate is indicated (N=3).

**(b)** Representative FACS plots of cord blood erythroid differentiation as a function of KIT/GPA on day 8 and CD49d/Band3 on day 9. The more mature cells are present in the indicated gate (N=3).

**(c)** Representative FACS analyses of erythroid differentiation of sh1-BID- and control- transduced CD34+ cells issued from bone marrow as a function of KIT/KPA and Band3/CD49d at the indicated time of the culture (N= 3).

**(d)** Representative FACS plots of GFP+ transduced cells as a function of PI and Annexin-V in the presence or not of the agonist of FasL mimetic antibody CH11 (N=4).

**Supplementary Figure 2. P18-tBID and P13-tBID are generated by activated caspase-10**

**(a)** Representative Western blot detection of caspase-10 after CD36+ cell sorting culture (day 0) and day 4 of the CD36 cells culture.

**(b)** To show that overexpression of wild type and mutant BID did not interfere with erythroid differentiation, BID vectors were expressed in BID knockdown erythroid cells (shBID-3’UTR) with co-transduction of both vectors (BID and shBID-UTR) performed at day 5 of the CD34 culture. A representative flow cytometry histogram of erythroid differentiation on day 8 of the CD36 culture after co-transduction of indicated P22-BID constructs (mCherry) and shRNA-GFP targeting BID (3’UTR). Graph on the right panels showing the percentage of mature cells (GPA^high^) in each experiment on day 8, and the percentage of live cells at day 7. The mean ± SD of four replicates shown, together with the p value in an unpaired two-tailed Student’s t test.

**(c)** The Coomassie blue-stained gel of the anti-Flag immunoprecipitation did not show protein bands. Gel slices corresponding to the 22 kDa, 18 kDa and 13kDa were cut and proteins from these slices were digested, peptides were extracted and analyzed by mass spectrometry.

**(d)** Mascot results for the N-terminus peptide of the 13kDa and the 18kDa BID protein form respectively. The upper panel shows the annotated spectrum, the lower table presents the identified peptide fragments (in red) among all possible fragments including those with two charges (superscript++), with NH2 loss (superscript*) or H2O loss (superscript 0)

**Supplementary Figure 3. Activated caspase-10 is required for erythroid terminal differentiation,**

**(a**) Representative Western blot detection of caspase-10, caspase-8 and caspase-3 performed on day 4 of GFP+ transduced cells with indicated shRNA, or in the presence of the CH11 antibody, showing the specificity of the shRNA targeting caspase-10 (N=3). _*_ activated caspase-8 or caspase-3.

**(b)**  Representative FACS plots of the apoptosis of GFP+ cells observed on day 4 and 7 of the CD36 culture, with sh1CASP10 or control transduction performed on day 5 of the CD34+ culture.

**(c)** Representative FACS analyses of erythroid differentiation of sh1-and sh2-caspase-10- and control- CD34+ transduced cells issued from bone marrow as a function of KIT/KPA and Band3/CD49d at the indicated time of the culture (N= 2).

**(d)** Graphs showing the percentage of transduced cells undergoing MOMP in the absence of apoptosis (left panel) and the percentage of the mature KIT^low^ GPA^high^ population (right panel) in the presence of antibodies blocking Fas and FasL (added on day 3 of the CD36 culture). The mean ± SD of three replicates is shown, together with the p value in an unpaired two-tailed Student’s t test.

**Supplementary Figure 4. P13-tBID induces terminal differentiation**

**(a**) Representative FACS and histograms plots of GPA and CD71 expression performed on P13-tBID-GFP+ cells and a control on day 3 of the cord blood CD34+ cells culture (N=4).

**(b)** Representative FACS analyses of erythroid differentiation of P13-tBID and control- CD34+ transduced cells issued from bone marrow as a function of Band3/CD49d at the indicated time of the culture (N= 3).

**(c)** Representative FACS quantification of GFP+ cells performed for indicated transduced cells at different time post infection. The gate indicates the percentage of GFP+ cells.

**Supplementary Figure 5. Specificity of the anti-PhBID-S65 antibody**

Flag-tagged forms of P18-tBID and P18-tBID-S64A-67A were transduced at day 5 of the CD34 culture. (left panel) A representative WB analyses of BID constructs phosphorylation after anti-FLAG immunoprecipitation in addition to total lysates (TL) showing that P18-tBID-S64A-S67A-FLAG phosphorylation was decreased compared to P18-tBID-FLAG (right panel) The graph gives the quantified band intensities of the ratio Ph-BID/ BID-FLAG. Values were normalized on P18-tBID phosphorylation. The mean ± SD of four replicates are indicated together with the p value in an unpaired two-tailed Student’s t test.jj

**Supplementary Figure 6. CK1α knock-down induces apoptosis and inhibits terminal differentiation.**

shCK1α or control transduction were performed at day 2 of the cord blood CD36 culture.

**(a)** Representative FACS plots of apoptosis of transduced shCK1α-GFP+ and control cells transduced cells performed at day 6 of the CD36 culture (N=5).

**(b)** Representative FACS plots of differentiation of GFP+ transduced cells as a function of KIT/GPA, observed on day 6 of the culture (N=5).

**(c)** Representative FACS analyses of erythroid differentiation of sh1-, sh2-shCK1α and control CD34+ transduced cells issued from bone marrow as a function of KIT/KPA and Band3/CD49d at the indicated time of the culture (N= 3)

**Supplementary Figure 7. Uncropped western blots**

**Supplementary Methods**

**Western blotting**

Primary erythroid cells were harvested on the indicated day, washed with PBS, and stained with propidium iodide (PI) and the annexin V-APC apoptosis kit or the annexin V-eFluor450 apoptosis kit (eBioscience), according to the manufacturer’s instructions. Live GFP+/annexin V-negative cells were then sorted (Sony SH800). Sorted cells were washed with PBS and lysed with Laemmli buffer at 90° for 10 min. Equal amounts of proteins (quantified with the Micro BCA^TM^ Protein assay kit (Thermo Scientific)) were separated by SDS electrophoresis on Tris/TRICINE 16.5% minigels (Biorad). Antibodies used were: rabbit polyclonal anti-BID (LSbio LS-C148235) at a 1/1000 dilution, rabbit polyclonal anti-BID (Santa Cruz) at a 1/500 dilution, rabbit polyclonal anti-PhBID-pSer65 (Thermo Fisher PA5-12555) at a 1/1000 dilution, rabbit polyclonal anti-PhBID-pSer65 (Abcam) at a 1/1000 dilution, mouse monoclonal anti-beta actin (Proteintech) at a 1/2000 dilution, rat monoclonal anti-HSC70 (Enzo Life Science) at a 1/2000 dilution, mouse monoclonal anti-caspase-10 (MBL) at a 1/500 dilution, mouse monoclonal anti-caspase-8 (MBL) at a 1/1000 dilution, goat polyclonal anti-CK1α (Santa Cruz) at a 1/1000 dilution, rabbit polyclonal anti-CK1 α (Abcam) at a 1/1000 dilution, mouse monoclonal anti-BAX (BD) at a 1/1000 dilution, or goat polyclonal anti-BAK1 (Thermo Fisher) at a 1/1000 dilution. Proteins were visualized with the Chemidoc XRS+ Imaging System (Biorad). Band intensities were determined with ImageLab software (Biorad) and normalized against actin or HSC70.

**Mass spectrometry**

The gel fragments were destained twice with a mixture of 100 mM ammonium bicarbonate (ABC) and 50% (vol/vol) acetonitrile (ACN) for 30 min at room temperature, dehydrated with 100% ACN, and then dried. Gel fragments were rehydrated with 25 mM ABC containing 10 mM DTT, and incubated for 1 h at 56 °C to reduce disulfide bonds. Proteins were then alkylated with 55 mM iodoacetamide in 25 mM ABC for 30 min in the dark at room temperature. Gel pieces were then washed twice with 25 mM ABC, dehydrated with 100% ACN, and dried. The pieces were rehydrated with 40 mM ABC containing 12.5 ng/μl sequencing-grade modified trypsin (Promega, USA) and 10% ACN, and incubated overnight at 37°C. After digestion, peptides were extracted twice from gel pieces with a mixture of 50% ACN – 5% formic acid (FA) and once with 100% ACN and dried.

Mass spectrometry (MS) analyses were performed on a Dionex U3000 RSLC nano-LC system coupled to a Q-Exactive Plus mass spectrometer (Thermo Fisher Scientific). Dried peptides were solubilized in 7 µL of 0.1% trifluoroacetic acid (TFA) containing 10% ACN. One microliter was loaded, concentrated and washed for 3 min on a C_18_ reverse phase precolumn (particle size: 3 µm; pore size: 100 Å; inner diameter: 75 µm; length: 2 cm; Thermo Fisher Scientific). Peptides were separated on a C_18_ reverse phase column (particle size: 2 µm; pore size: 100 Å; inner diameter: 75 µm; length: 25 cm; Thermo Fisher) with a 1-hour gradient starting with 99% of solvent A containing 0.1% formic acid (FA) in H_2_O and ending in 40% of solvent B containing 80% can and 0.085% FA in H_2_O. The mass spectrometer acquired data throughout the elution process, and operated in a data-dependent configuration with full MS scans acquired with the Orbitrap, followed by up to 10 MS/MS HCD fragmentations in the Q-Exactive Plus for the most abundant ions detected. The resolution was set to 70,000 for full scans at AGC target 3.0x10^6^ within a 100 ms maximum injection ion time (MIIT). The MS scans ranged from 400 to 2000 Th. The precursor selection window was set to 4 Th, and MS/MS scan resolution was set to 17,500 with an AGC target 1.0x10^5^ and a 100 ms MIIT. The HCD collision energy was set to 30%. Dynamic exclusion was set to 15 s. Proteome Discoverer software (version 1.4, Thermo Fisher) was used to generate .mgf files. The mass spectrometry data were analyzed with Mascot software (version 2.5, Matrix science) using the Uniprot human protein database (Release dated July 2017; 20,289 sequences) from the SwissProt databank. The enzyme specificity was semi-trypsin, and a single missed cleavage was tolerated. The precursor mass tolerance was set to 4 ppm and the fragment mass tolerance was set to 20 mmu. Carbamidomethylation of cysteine residues was set as a constant modification, and the oxidation of methionine residues was set as a variable modification.

**Plasmid constructs and cloning**

Full-length human BID was PCR-amplified (Accuprime^TM^ *Pfx* DNA polymerase, Invitrogen) from the pLenti-GIII-CMV-hBID vector (ABM inc), and cloned into a pcDNA3.1 vector (Thermo Fisher) for further mutagenesis or a pLVX-EF1-IRES-ZsGreen vector (Clontech) using EcoR1 and Xba1 restriction sites. Point mutations in BID were generated using the Geneart Site-Directed Mutagenesis kit (Invitrogen), according to the manufacturer’s recommendations. P18-tBID, P18-tBID:D60A, P18-tBID:D75A, P18-tBID:D60A-D75A, P18-tBID:S64-67A, P18-tBID:S64-67D and P13-tBID constructs were PCR-cloned into the pLVX-EF1-IRES-ZsGreen or pLVX-EF1-IRES-mCherry vectors using EcoR1 and Xba1 restriction sites. A FLAG-tag was inserted into the C-terminal end of BID constructs by PCR amplification and then cloning into the pLVX-EF1-IRES-ZsGreen vector via EcoR1 and BamH1 restriction sites. All constructs were checked by Sanger sequencing.

|  | |
| --- | --- |
| Primers for full length BID cloning:  Forward: 5’ gcgGAATTCCCatggactgtgaggtcaacaacg  Reverse:5’ctcTCTAGAtcagtccatcccatttctgg |  |
| Primer for P18-tBID cloning: Forward:5’TATCTTGAATTCATGGCACTGGGCCACGAGCTG |  |
| Primer for P13-tBID cloning: Forward: 5’ TATCTTGAATTCATGTCTGAAAGTCAAGAAGAC |  |
| S64-67D mutagenesis: Forward: 5’agACTGATGGCAACCGCGACAGCCACGACcgcttgggaagaatag |  |
| S64-67A mutagenesis: Forward: 5’GATGGCAACCGCGCCAGCCACGCCcgcttgggAAGAATAG |  |
| D60A mutagenesis: Forward: 5’-gagctgcagactgctggcaaccgcagc |  |
| D75A mutagenesis: Forward: 5'-ttgggaagaatagaggcagcttctgaaagtcaagaagac |  |
| Flag C-terminal cloning: Reverse: 5’ CTCTCGGATCCTTACTTGTCGTCATCGTCTTTGTAGTCgtccatcccatttctggctaa  The shRNA constructs were obtained from the Mission shRNA collection (Sigma Aldrich): |  |
| shBID-3UTR TRCN0000312688 : ATGTCCATTTACACGTATTTG |  |
| Sh1RNA-1 targeting BID TRCN0000062709 : CTTTCACACAACAGTGAATTT |  |
| shRNA-2 targeting BID TRCN0000312746  CCGGGAAGACATCATCCGGAATATTCTCGAGAATATTCCGGATGATGTCTTCTTTTTG |  |
| shRNA-3 targeting BID TRCN0000327898  CCGGGTGAGGAGCTTAGCCAGAAATCTCGAGATTTCTGGCTAAGCTCCTCACTTTTTG |  |
| shRNA-1 targeting CASP10 TRCN0000355598 CCGGCGTGAGAAGCTTCTGATTATTCTCGAGAATAATCAGAAGCTTCTCACGTTTTTG |  |
| shRNA-2 targeting CASP10 TRCN0000003585 CCGGAGGACAGACAAGGAACCCATACTCGAGTATGGGTTCCTTGTCTGTCCTTTTTT |  |
| shRNA-1 targeting CK1α TRCN0000199710 CCGGGCCACAGTTGTGATGGTTGTTCTCGAGAACAACCATCACAACTGTGGCTTTTTTG |  |
| shRNA-2 targeting CK1α TRCN0000196743 CCGGGCATCTAAAGTGAAGACTTAACTCGAGTTAAGTCTTCACTTTAGATGCTTTTTTG |  |
| shRNA-3 targeting CK1α TRCN0000010990 CCGGGCCTGCTTAATTGTGCTAGAACTCGAGTTCTAGCACAATTAAGCAGGCTTTTT |  |
