# Supplementary figures and images for: Role of Caspase-10-P13tBID axis in erythropoiesis regulation
Source: Cell Death Differ. 2022 Oct 6;30(1):208–20. doi: 10.1038/s41418-022-01066-0 (PMC9883265; doi:10.1038/s41418-022-01066-0)

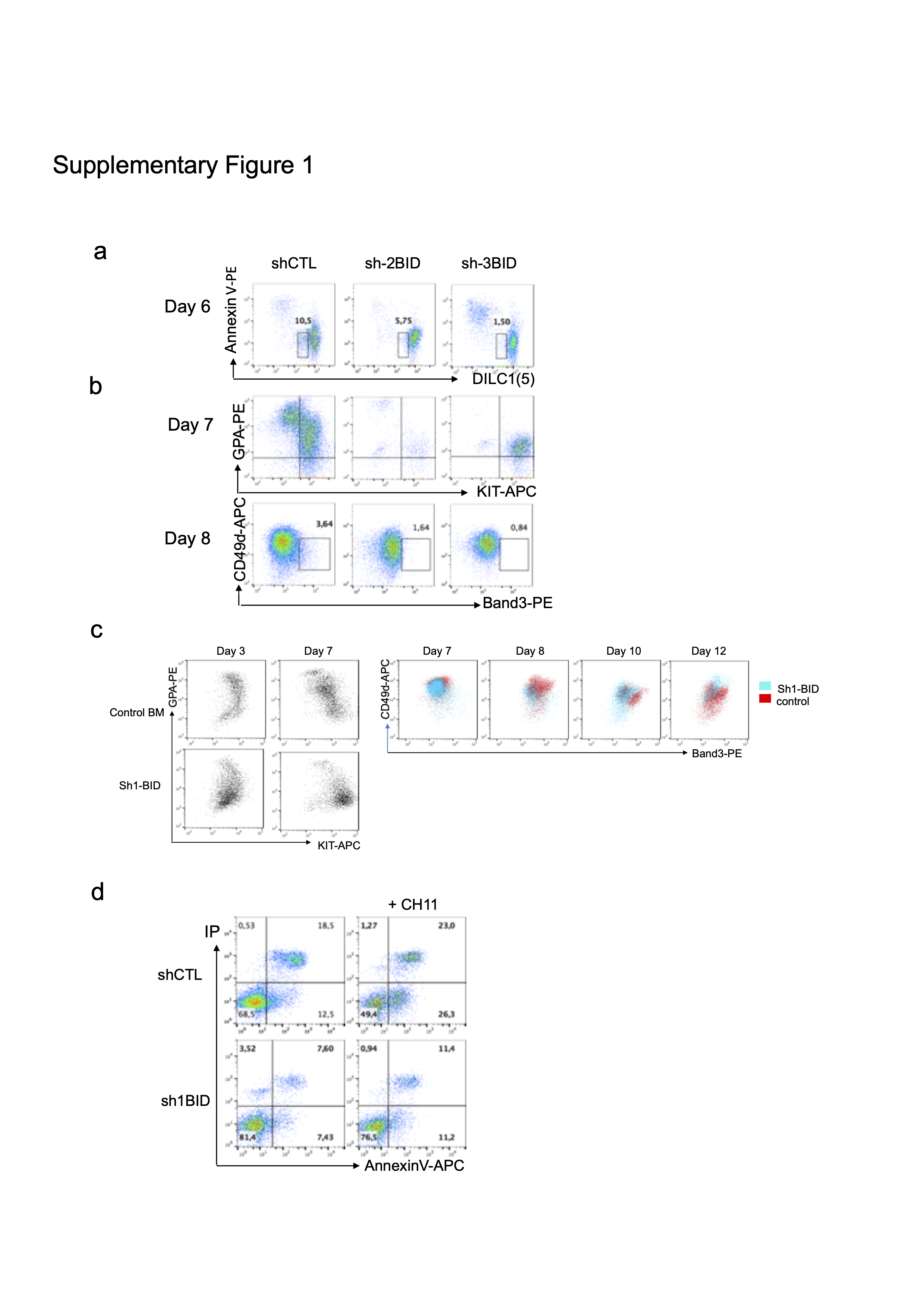

Supplement: Supplementary file 2 — supplementary Fig S1 [file 41418_2022_1066_MOESM2_ESM.tif]

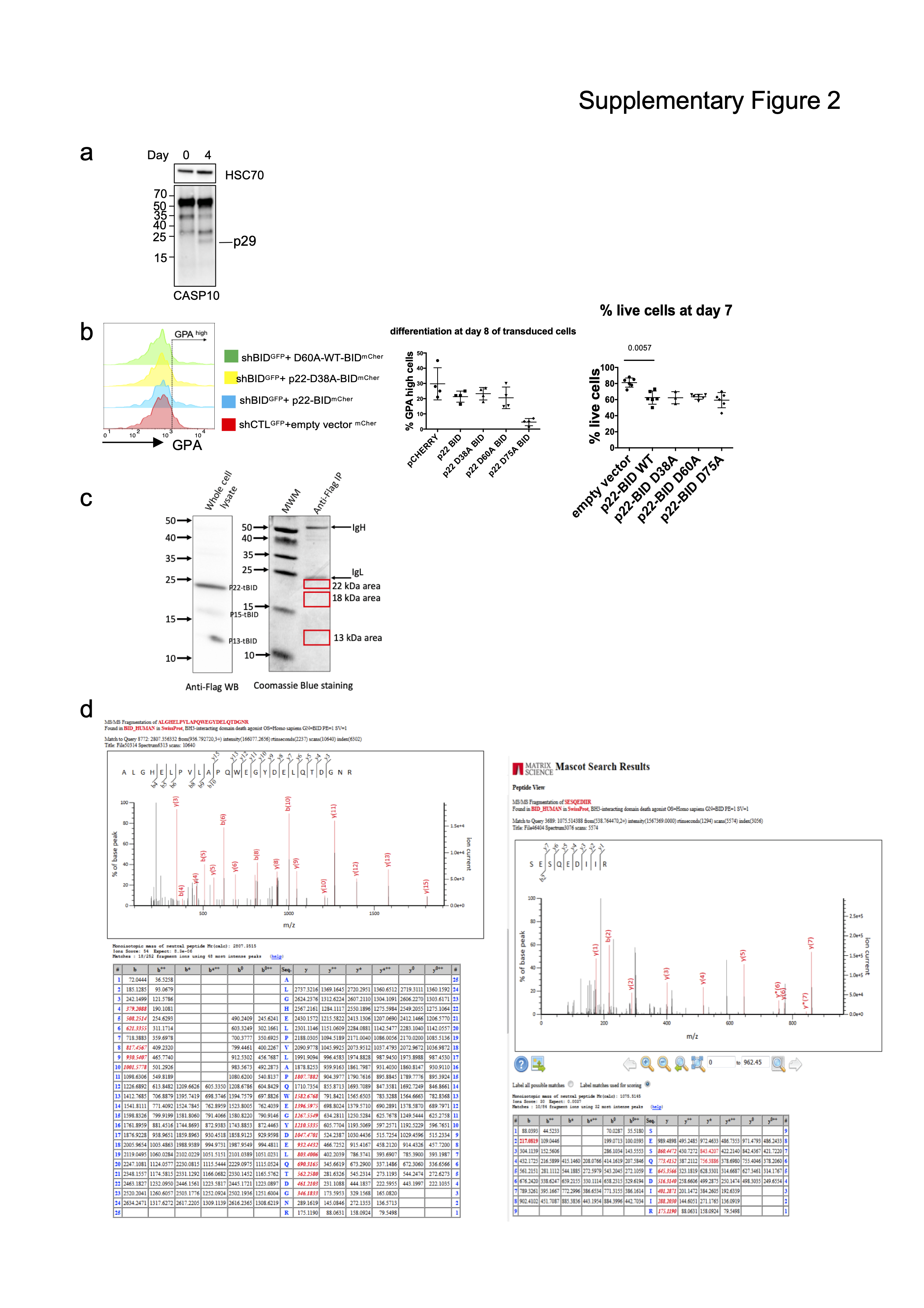

Supplement: Supplementary file 3 — supplementary Fig S2 [file 41418_2022_1066_MOESM3_ESM.tif]

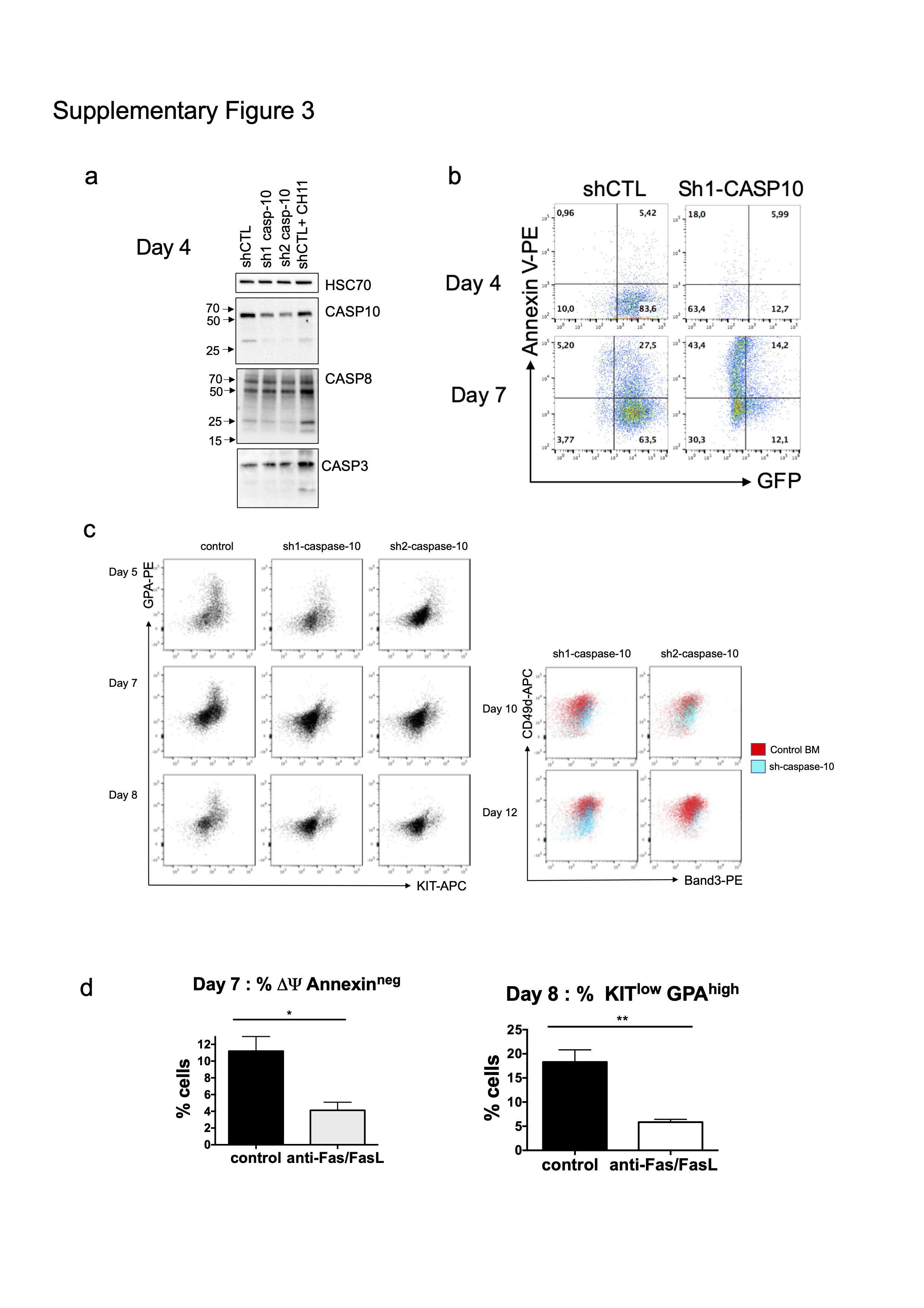

Supplement: Supplementary file 4 — supplementary Fig S3 [file 41418_2022_1066_MOESM4_ESM.tif]

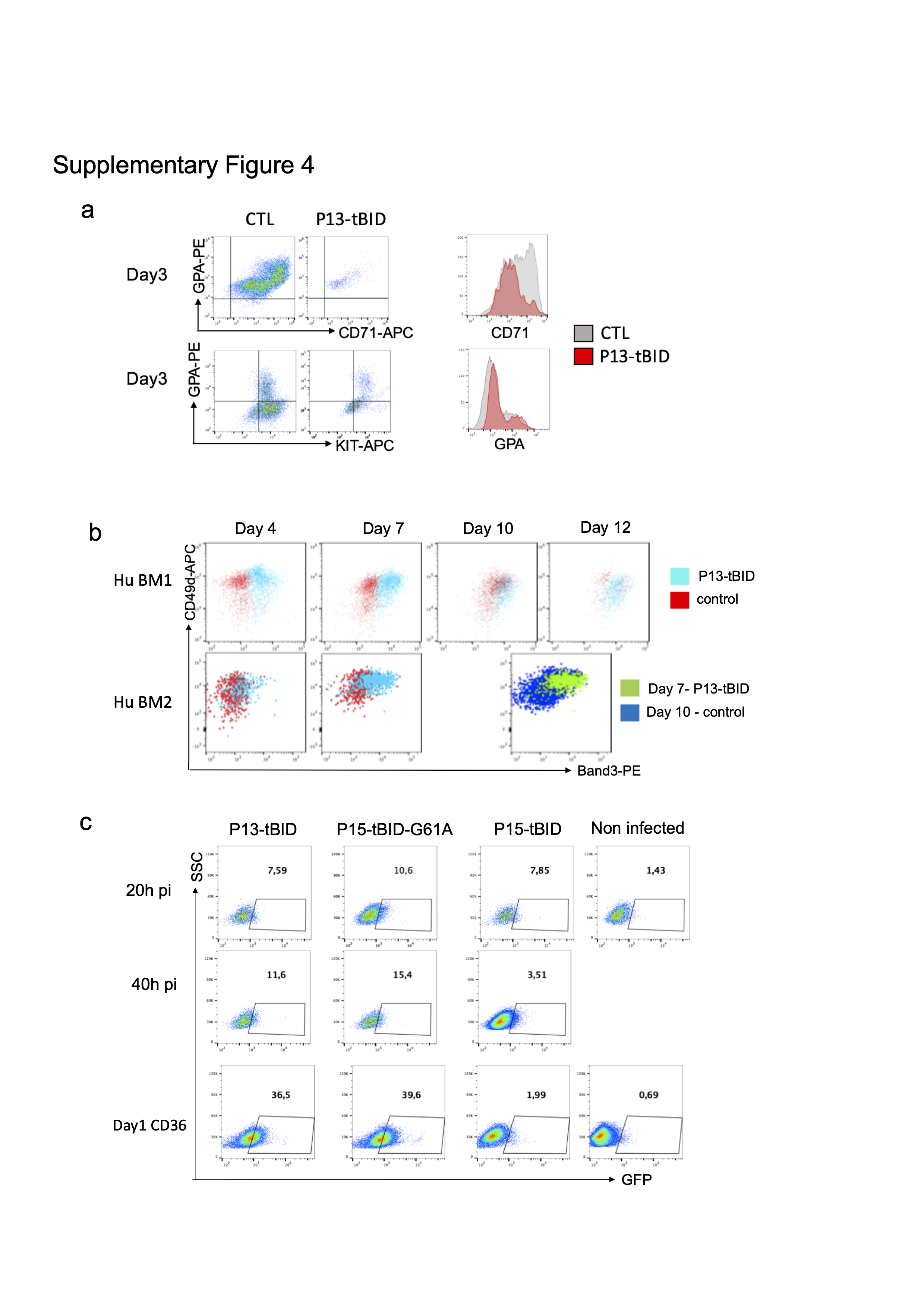

Supplement: Supplementary file 5 — supplementary Fig S4 [file 41418_2022_1066_MOESM5_ESM.tif]

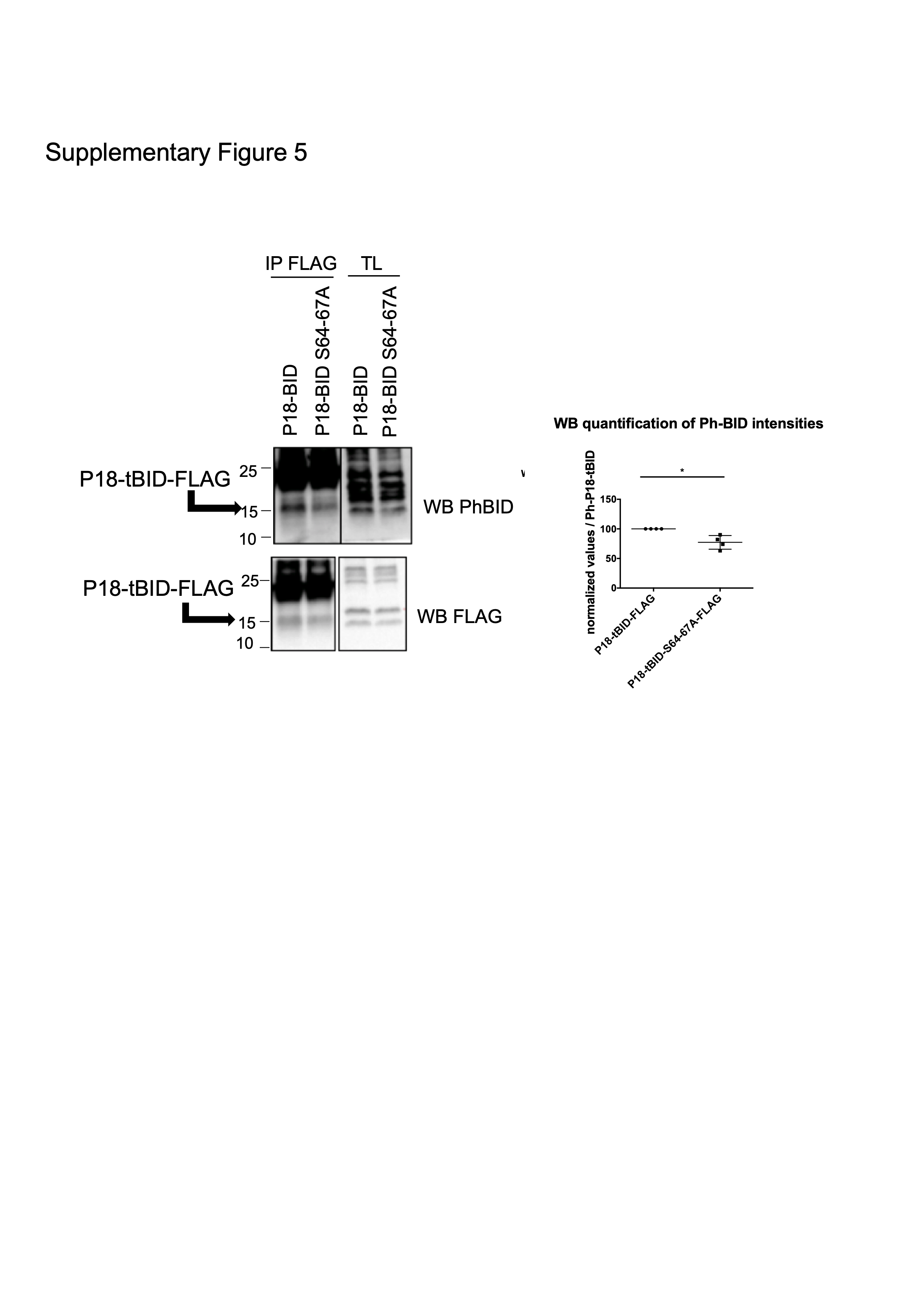

Supplement: Supplementary file 6 — supplementary Fig S5 [file 41418_2022_1066_MOESM6_ESM.tif]

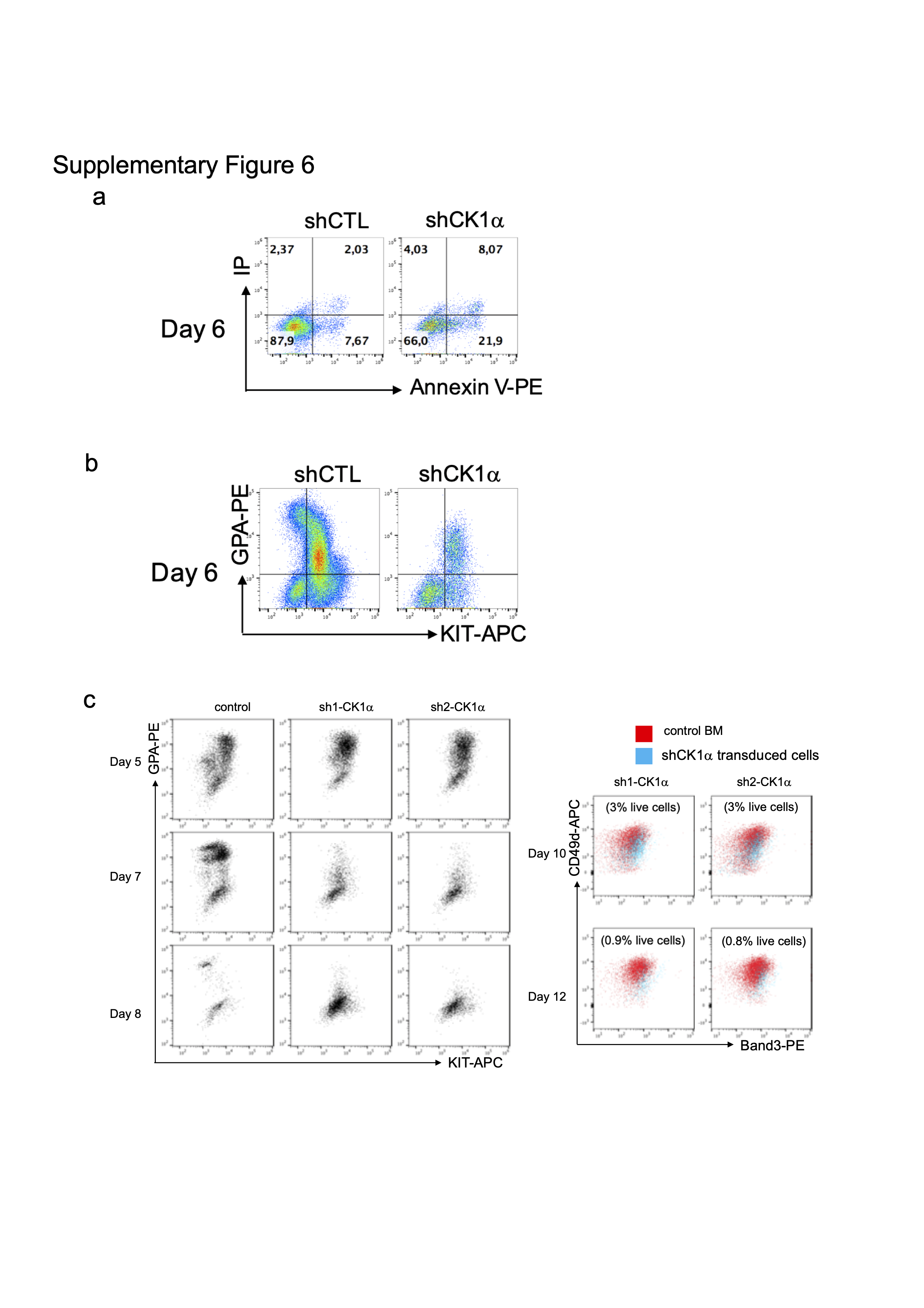

Supplement: Supplementary file 7 — supplementary Fig S6 [file 41418_2022_1066_MOESM7_ESM.tif]

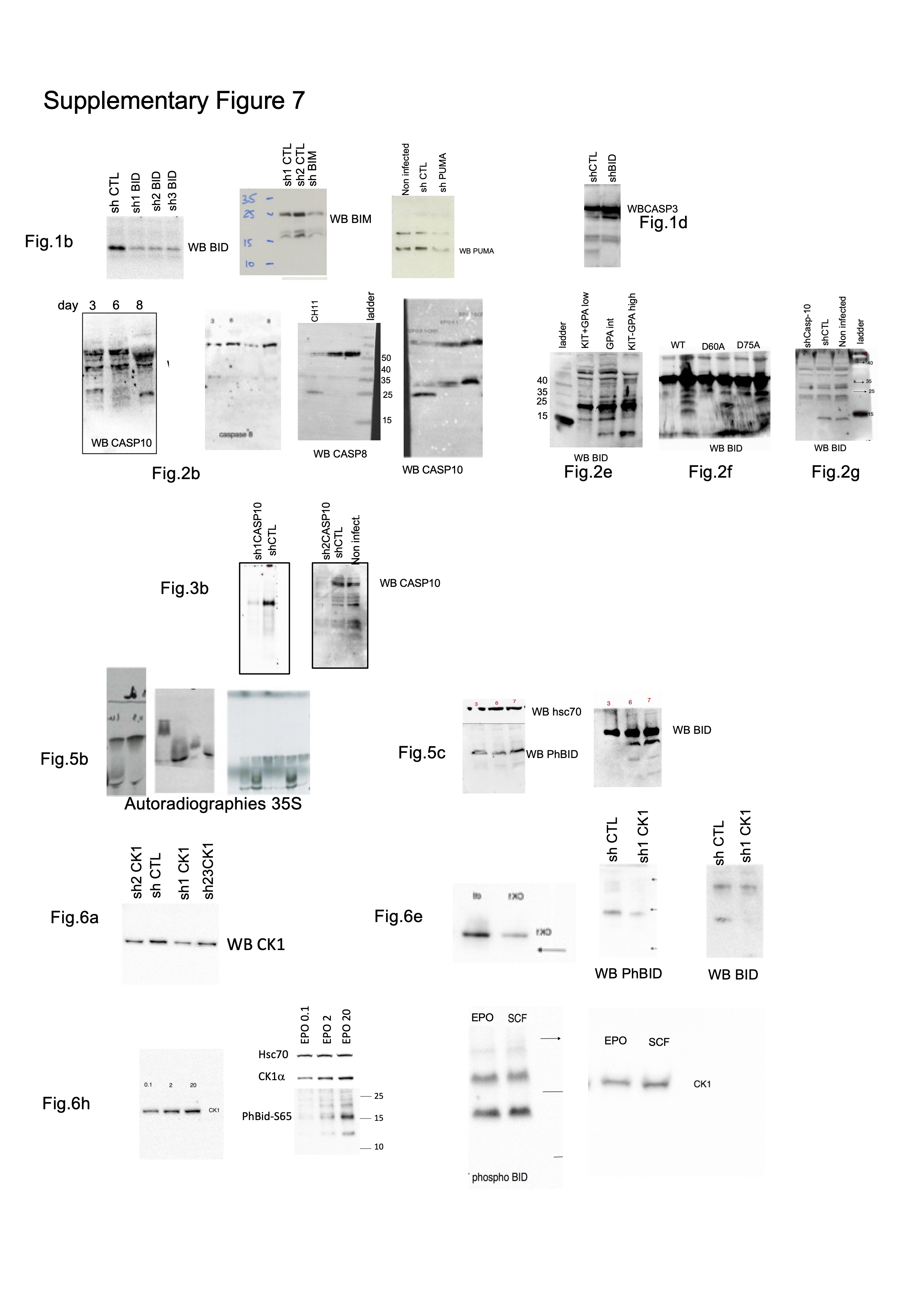

Supplement: Supplementary file 8 — supplementary Fig.S7 - uncropped western blots [file 41418_2022_1066_MOESM8_ESM.tif]
